# Mindful Eating: A Deep Insight Into Fructose Metabolism and Its Effects on Appetite Regulation and Brain Function

**DOI:** 10.1155/jnme/5571686

**Published:** 2025-04-21

**Authors:** Gabriela Vanessa Flores Monar, Camila Sanchez Cruz, Ernesto Calderon Martinez

**Affiliations:** ^1^Department of Endocrinology, Albany Medical Center Endocrinology Group, Albany, New York, USA; ^2^Faculty of Medicine, Universidad Nacional Autónoma de México, Coyoacán, Ciudad de México, Mexico

**Keywords:** appetite, brain, fructose, fructose metabolism, high-fructose diet, hypothalamus, neuroinflammation

## Abstract

Fructose, a common sweetener in modern diets, has profound effects on both metabolism and brain function, primarily due to its distinct metabolic pathways. Unlike glucose, fructose bypasses critical regulatory steps in metabolism, particularly the phosphofructokinase-1 (PFK-1) feedback inhibition, leading to uncontrolled metabolism and increased fat storage. This review delves into the metabolic consequences of fructose consumption, including its limited role in directly stimulating insulin secretion, which affects satiety signaling and contributes to increased food intake. The small intestine initially helps metabolize ingested fructose, shielding the liver and brain from excessive exposure. However, when consumed in excess, particularly in diets high in processed foods, this protective mechanism becomes overwhelmed, contributing to metabolic disorders such as insulin resistance, obesity, and fatty liver disease. The review also explores fructose's impact on the brain, with a focus on the hippocampus, a key region for memory and learning. Chronic high fructose intake has been linked to mitochondrial dysfunction, increased production of reactive oxygen species (ROS), and neuroinflammation, all of which contribute to cognitive decline and impairments in memory and learning. Additionally, fructose-induced alterations in insulin signaling in the brain are associated with increased risk for neurodegenerative diseases. These findings underscore the potential long-term neurological consequences of excessive fructose intake and highlight the need for further human studies to assess the full spectrum of its effects on brain health. Addressing the rising consumption of fructose, particularly in processed foods, is essential for developing targeted strategies to mitigate its adverse metabolic and cognitive outcomes.

## 1. Introduction

Throughout evolution, mammals presumably used excessive fructose intake and metabolism as a vital survival purpose to store energy to ensure availability in times of scarcity [1]. This suggests that high fructose ingestion turns the body into a low-energy mode in which there is reduced adenosine triphosphate (ATP) production and usage while encouraging hunger to promote further food-seeking behavior [2–5]. Additionally, fatty acid oxidation, lipolysis, and glycogenolysis are inhibited to store glycogen and fat in the liver [6–8]. Interestingly, this survival mode mechanism is unique for fructose, as glucose has opposite effects. It can be deduced that glucose is an essential fuel for immediate energy demands, and fructose ensures energy storage for future needs. However, in contemporary times, with the dramatically increased consumption of processed foods, high-fructose diets, and a sedentary lifestyle, this once beneficial process has resulted in detrimental effects on the body and brain function [1, 9].

In the past centuries, the fructose diet in humans consisted of natural fructose-containing foods like fruit, vegetables, and honey, but with the development of industrial and manufactured products in the early 20th century, the sources of fructose changed, and now they mostly appear as added sugars [10–12]. These added sugars, processed or refined sugars, have sucrose and high-fructose corn syrup (HFCS) as their leading exponents [13–16].

Fructose is a strong lipid-promoting sugar as a precursor of fatty acid synthesis without feedback inhibition through the phosphofructokinase (PFK) pathway [17, 18]. It promotes fat storage by increasing the production of fatty acids and decreasing fatty acid oxidation [19]. Additionally, fructose affects insulin signaling and may lead to insulin resistance [20]. Research studies also corroborate that ingesting high fructose can increase the number of active oxygen species, as fructose is heavily involved in inflammatory and oxidative damage [21, 22].

This review examines how increased fructose consumption can induce changes in the hippocampus and other brain regions associated with regulating the appetite-reward system. It also explains how its unchecked metabolism can cause ATP depletion, leading to neuroinflammation, mitochondrial dysfunction, and oxidative stress in the brain, which in turn leads to memory and cognitive impairment [9, 23, 24].

## 2. Fructose Metabolism

Fructose and glucose share an identical molecular composition, C6H12O6, and provide the same caloric density of 4 kcal/g. However, substantial differences are observed in their respective physiological influences and taste perceptions. Fructose exhibits a sweetness index value of 1.7, significantly higher than glucose's index of 0.75. Additionally, fructose has a lower impact on postprandial glycemia, as evidenced by its glycemic index of 23, compared to the glycemic index of 100 attributed to glucose [25, 26]. A worth mentioning feature is that fructose ingestion, unlike glucose, only weakly impacts the circulating levels of insulin [27], which acts to increase the feeling of satiety [28]. Fructose also decreases glucagon-like peptide 1 (GLP-1)24, a satiety hormone, and does not attenuate increasing levels of the appetite-stimulating hormone ghrelin [29, 30]. All these features could trigger a less substantial satiety response and, therefore, an increase in overall food consumption [31].

**Figure 1:**
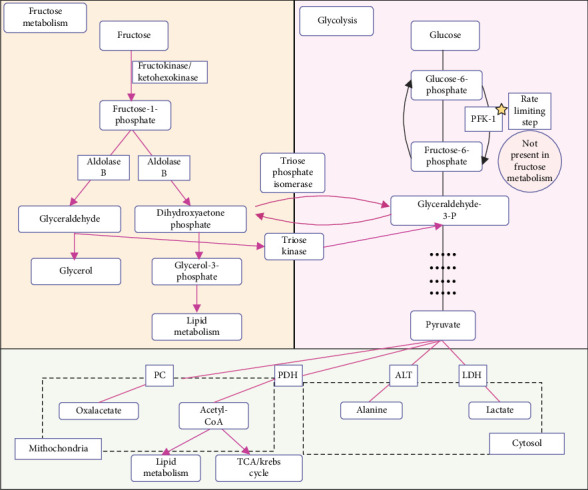
Comparative metabolism of fructose and glucose. ALT = alanine transaminase, LDH = lactate dehydrogenase, PC = pyruvate carboxylase, PDH = pyruvate dehydrogenase, PFK-1 = phosphofructokinase-1, TCA = tricarboxylic acid cycle.

Fructose metabolism is a process that lacks the regulatory steps seen in the metabolic glucose pathway, mainly the irreversible step of the conversion of fructose-6-phosphate (F6P) to fructose-1,6-bisphosphate by PFK-1 [32]. In fructose catabolism, once inside the cell, fructose undergoes rapid phosphorylation by fructokinase or ketohexokinase (KHK) to form fructose-1-phosphate (F1P), and it bypasses the control of the PFK-dependent step. In the absence of such negative feedback regulation, F1P undergoes further catabolism into the glycolytic intermediate, dihydroxyacetone phosphate (DHAP), and glyceraldehyde [33, 34]. In the final step of the pathway, both DHAP and glyceraldehyde can phosphorylate to form glyceraldehyde-3-phosphate (G3P) [32]. DHAP and G3P act similarly to the glycolytic intermediates and enter gluconeogenesis. They can also be converted to glycerol-3-P and form methylglyoxal (MA) and free fatty acids (FFA) and triglycerides (TG) via de novo lipogenesis (DNL) or be catabolized in the glycolytic downstream pathway into different compounds like lactate, acetyl-CoA, alanine, and oxalacetate [32, 33, 35] (Figure 1). The overproduction without feedback inhibition leads to an overproduction of metabolic byproducts typically involved in glycolysis, fatty acid synthesis, and triglyceride formation, contributing to metabolic disturbances [32–34, 36–38].

## 3. Transport of Fructose From the Small Intestine to the Liver

**Figure 2:**
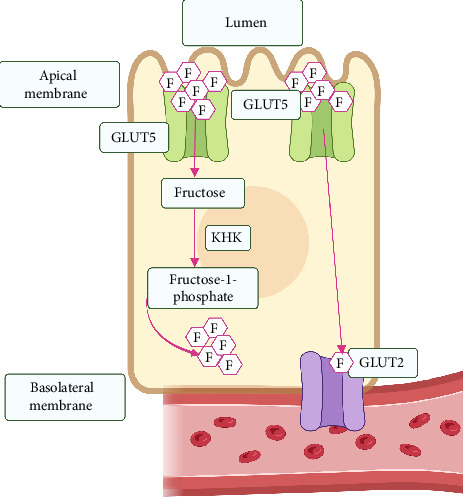
Intestinal absorption of fructose. Fructose molecules (F) from the intestinal lumen are transported across the apical membrane by GLUT5 transporters. Once inside the intestinal cell, fructose is phosphorylated by the enzyme ketohexokinase (KHK) to fructose-1-phosphate and its respective fructose metabolites (FM). Some of the fructose molecules are also channeled directly into the bloodstream through the GLUT2 transporters located at the basolateral membrane, highlighting the dual pathway of fructose absorption.

GLUT5 and GLUT2 are members of the facilitative glucose transporter family and serve as the primary transporters for fructose in the body [39, 40]. Fructose from the diet is absorbed in the apical membrane of the small intestine through GLUT5 [41]. At the same time, GLUT2 plays a crucial role as the primary fructose transporter in the basolateral membrane, moving fructose from the cytosol into the bloodstream [40, 42–44], as shown in Figure 2.

The small intestine is crucial in the metabolic process of fructose, as it converts it into glucose and other metabolites [32, 33, 35], thus protecting the liver from direct fructose exposure and consequent damage [45]. In a research study, mice's fructose and glucose metabolism pathways were assessed by labeling them with isotope tracers and analyzing them with mass spectrometry. In the case of orally administered labeled fructose, a significant accumulation of F1P was observed in the small intestine rather than the liver [46]. When physiological doses of fructose are ingested, the small intestine clears almost 90% of ingested fructose. Still, when higher doses are administered, they overwhelm the small intestine metabolism and pass directly to the portal vein, leading to spillage into the liver and colonic microbiota and inducing subsequent toxicity [45, 47]. In such scenarios, excess fructose that reaches the liver contributes to fat formation, while fructose catabolized by gut microbiota induces alterations in composition and dysbiosis [47, 48].

In normal circumstances, as mentioned, fructose is transported from the basolateral membrane of enterocytes to the systemic circulation by GLUT2 and then reaches the liver [32]. The remaining fructose can reach the brain in small amounts, evidenced by studying the fate of fructose in mice with labeled fructose with mass spectrometry [45]. These findings go in hand with the findings of GLUT5 in the blood–brain barrier, choroid plexus, and microglia, suggesting a feasible fructose metabolism in these organs [49–51]. Animals lacking GLUT5 exhibit fructose intolerance and even death due to their inability to absorb fructose. Specifically, Barone et al. [52] showed that GLUT5 knockout (KO) mice maintained normal healthy phenotypes without dietary fructose consumption. However, when exposed to increased dietary fructose intake, they developed severe hypovolemic shock and died after 7–10 days.

**Figure 3:**
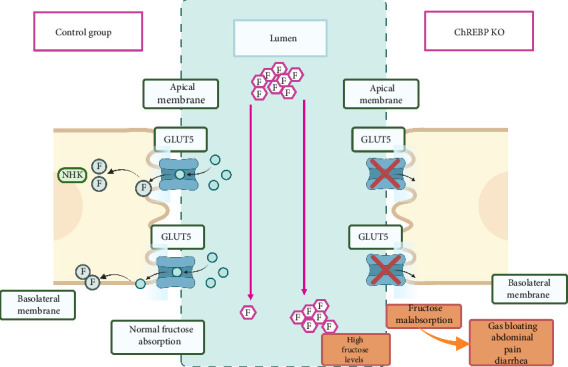
Differential fructose absorption in normal vs. ChREBP KO Mice. F = fructose, FM = fructose metabolites.

It is known that fructose transporter GLUT5 and the KHK enzymes are involved in the upregulation of the enzymes involved in fructose catabolism, and one of its main goals is to purposely increase the ability of the small intestine to respond to an anticipated increase in ingested glucose [53]. Carbohydrate response element-binding protein, also known as ChREBP, is another transcription factor linked as a critical regulator of lipogenesis, glycolysis, gluconeogenesis, triglyceride formation, and fructose metabolism. It induces gene expression as a response to consumption of carbohydrates [54–60] in an insulin-independent process [61]. ChrREBP also increases hepatic KHK and upregulates GLUT5 gene expression after increased fructose intake [62]. Also, it increases the expression of fructolytic enzymes such as triokinase, LDH, and ALDOB [63]. Accordingly, studies have shown that ChREBP KO mice showed hypothermia, quick weight loss, and a moribund state when subjected to sucrose or fructose diets [55, 63]. This fructose intolerance was proposed to be caused by the liver's downregulation of enzymes like KHK and ALDOB. Consequently, high fructose concentrations in the small intestine cause water to enter the lumen and rapidly release contents into the colon. Finally, bacteria in the colon ferment the unabsorbed fructose and may cause bloating, gas, and diarrhea [56] (Figure 3).

## 4. Metabolic Effects of Fructose From Whole Fruits vs. Processed Sources

Research has shown that the metabolic effects of fructose vary depending on its source. A cross-sectional study of 41,714 participants found that fructose from sugar-sweetened beverages (SSBs) was linked to unfavorable biomarker profiles, including increased levels of inflammatory markers (CRP, IL-6, tumor necrosis factor (TNF)-R1, TNF-R2, and leptin), insulinemic/glycemic markers (C-peptide and HbA1c), and lipid markers (total cholesterol, LDL-C, triglycerides, and the triglyceride-to-HDL-C ratio). Fructose from fruit juice also correlated with higher concentrations of C-peptide, HbA1c, and triglycerides, alongside lower adiponectin levels. In contrast, fructose from whole fruit was associated with lower levels of C-peptide, CRP, IL-6, leptin, and total cholesterol, as well as higher TNF-R2 levels [64]. Another cross-sectional analysis of 3981 individuals further confirmed that fructose intake from SSBs and juice was associated with higher intrahepatic lipid content, while fructose from fruit did not have this effect [65]. The physical form of fructose plays a key role in shaping its metabolic effects. Whole fruits are rich in fiber, which slows digestion and regulates fructose absorption, helping to prevent rapid spikes in blood sugar and lipid production. In contrast, fructose from SSBs and fruit juices is absorbed more quickly, leading to faster hepatic fructose exposure and higher rates of DNL. While fruit juices retain some beneficial compounds, their lack of fiber makes them less effective in regulating fructose absorption and mitigating metabolic disturbances [66]. Additionally, the vitamins, antioxidants, and phytochemicals in fruit contribute to reduced systemic inflammation and overall metabolic benefits [67]. In contrast, fructose from HFCS, SSBs, and fruit juices is typically consumed in larger quantities, overwhelming the small intestine's metabolic capacity. This results in excessive fructose reaching the liver, contributing to hepatic fat accumulation, insulin resistance, dyslipidemia, and nonalcoholic fatty liver disease [68–70]. Furthermore, the unregulated metabolism of HFCS and liquid fructose increases uric acid production and inflammatory markers, heightening cardiovascular risk [64, 71].

## 5. Brain Areas Involved in Food Intake and Satiety

**Figure 4:**
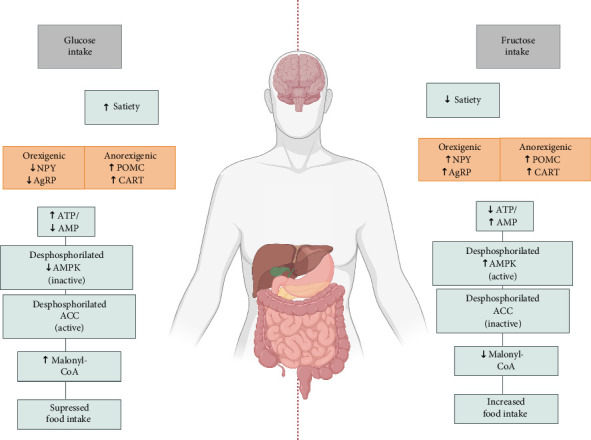
Effects of fructose vs. glucose intake on satiety and metabolic pathways and energy regulators. Effects ACC = acetyl-CoA carboxylase, AgRP = agouti-related peptide, CART = cocaine- and amphetamine-related transcript, NPY = neuropeptide Y, POMC = pro-opiomelanocortin.

The hypothalamus is known to play a key role in the homeostasis of food intake as it receives signals from the gastrointestinal tract through the brainstem, and it is also connected to close areas involved in maintaining the energy balance of the body [72]. Five hypothalamic nuclei have been described in association with food intake and appetite regulation: the ventromedial, lateral, dorsomedial, arcuate nuclei, and paraventricular nucleus [73]. Within the arcuate nucleus, first-order neurons function as metabolic sensors, integrating signals from peripheral sources and possessing antagonistic effects on food intake. Specifically, one group of neurons co-expresses agouti-related peptide (AgRP) and neuropeptide Y (NPY). It projects their effects to second-order neurons in the paraventricular nucleus to trigger orexigenic effects. In contrast, another subset of neurons expressing pro-opiomelanocortin (POMC) cocaine and amphetamine–related transcript (CART) project to second-order neurons localized in the lateral hypothalamic area, leading to anorexigenic effects or inhibition of food intake [74–77]. The sensitivity of these neuron types to fluctuations in hormone levels plays a vital role in modulating food intake [78, 79] (Figure 4).

The regulation of appetite and satiety is a multifaceted process involving a complex network of brain regions, and it is also subjected to influences of the hedonic reward of food, palatability, environment, and emotional state [80]. In the hypothalamus, second-order neurons send signals to some regions of the limbic system like the amygdala (handles emotions), hippocampus (important for memory), insula (involved in perception), striatum (related to reward and motivation), and the orbitofrontal cortex (which plays a role in decision making) [80].

Nevertheless, this field of study faces challenges due to the variability in research findings. Discrepancies in the specific brain areas involved in inducing hunger and satiety are common, primarily attributed to different study designs, methods used to administer nutrients, and the stimuli influencing food intake. To clarify these differences, a recent meta-analysis, which examined data from about 212 participants in different studies, offered new insights. This analysis showed that the amygdala, hippocampus, insula, and orbitofrontal cortex correlated with appetite regulators. The hypothalamus, caudate nucleus, putamen, thalamus, and anterior cingulate cortex worked as satiety regulators. Interestingly, the insula and orbitofrontal cortex contributed to both hunger and satiety regulation, emphasizing the interconnected nature of these systems [81].

## 6. Effects of Fructose and Glucose on Hypothalamic Energy Regulators: ATP, ACC, Malonyl-CoA, and AMPK

The body's energy levels dictate different signals for hormones and neurons to be activated or inhibited. For example, when the reserve of lipids is high, leptin levels in the circulation increase [82]. Leptin is a hormone that exerts an anorexigenic effect and reduces food intake by increasing the POMC/CART neurons and inhibiting the NPY/AgRP neurons. Likewise, insulin plays a significant role in food intake regulation. After food is ingested, insulin levels rise and exert anorexigenic stimuli. These hormones also are interconnected with intestinal peptides, which also regulate appetite. Specifically, GLP-1, cholecystokinin (CCK), and PYY provide satiety; on the contrary, ghrelin stimulates food intake [26, 29, 83–87].

In the same way, acting like a fuel meter for cells, when the body experiences excess energy, ATP increases and AMP decreases [88]. AMP is an activator of AMPK. Therefore, a decrease in AMP leads to the inactivation or dephosphorylation of AMPK. Similarly, AMPK catalyzes the activation or phosphorylation of acetyl-CoA carboxylase (ACC), a key regulator of fatty acid biosynthesis. Therefore, a decrease in AMPK causes the dephosphorylation of ACC. In the case of ACC, dephosphorylation leads to its activation. The activation of ACC, particularly in the hypothalamus, is prominent during a positive energy balance, leading to increased production of malonyl-CoA, which is the reaction product of ACC and is known for suppressing food intake [89, 90] (Figure 4).

**Figure 5:**
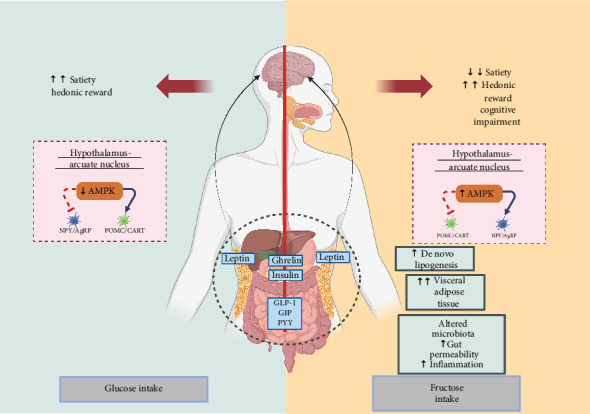
Differential effects of glucose and fructose intake on satiety, hedonic reward, and metabolic pathways. AMPK, AMP-activated protein kinase; NPY, neuropeptide Y; AgRP, agouti-related peptide; POMC, Pro-opiomelanocortin; CART, cocaine- and amphetamine-regulated transcript; GLP-1, glucagon-like peptide 1; GIP, gastric inhibitory polypeptide; PYY, peptide YY.

In the hypothalamus, various neuropeptides are expressed according to the level of malonyl-CoA. As previously mentioned, some orexigenic neuropeptides that promote hunger are the NPY and AgRP, and some essential anorexigenic peptides are the α-melanocyte-stimulating hormone (α-MSH), POMC, and CART, which decrease appetite [90–94]. Signals of leptin and insulin inhibit AMPK activity, contributing to appetite regulation [94, 95]. Thus, from intracerebroventricular glucose administration in rodents, which involves this hormone, suppresses food intake. The limited ability of fructose to stimulate satiety hormones like leptin and insulin results in AMPK activation and minimally excites POMC neurons while keeping NPY/AgRP neuron signals active, leading to lower satiety than glucose and consequently more food intake [89, 95–98]. This mechanism is illustrated in Figure 5, which highlights the opposing effects of glucose and fructose on hypothalamic AMPK activity and neuropeptide regulation.

In a study conducted by Wolfgang et al., [90] it was shown that immediately after the administration of glucose into the central nervous system of fasted mice, the levels of malonyl-CoA rose, along with an increased expression of the anorexigenic neuropeptides CART and α-MSH and the decreased expression of the orexigenic ones as NPY and AgRP in the hypothalamus. And within 30 min of glucose administration, food intake was visibly reduced when mice were given access to food.

It was previously proven [90] that 2-deoxyglucose (2-DG) inhibited the conversion of glucose to malonyl-CoA in the hypothalamus, as 2-DG is a potent inhibitor of glucose-6-phosphate and the glycolytic pathway. Accordingly, to study the different effects of central glucose and fructose on hypothalamic malonyl-CoA and ensure the impact of fructose before being converted to glucose in the brain, Cha et al. [89] conducted research in which 2-DG was injected into mice before the administration of fructose. The results were that fructose, administered centrally, caused a decrease in ATP in the hypothalamus, an increase in AMP and activation of AMPK, and a reduction in the levels of hypothalamic malonyl-CoA, and glucose showed the exact opposite effects, both measured in the same time frame of about 10–20 min after administration. Their study [89] also showed that centrally administered glucose caused an increase in POMC and CART mRNA levels and decreased levels of hypothalamic AgRP and NPY mRNAs. In contrast, centrally administered fructose decreased the levels of POMC mRNA, which translates to fructose inducing an orexigenic effect and had almost no impact on the levels of NPY, AgRP, or CART levels.

Therefore, fructose metabolism takes a unique path, different from the tightly regulated steps of glucose metabolism. Specifically, it skips the PFK feedback inhibition, a critical regulatory step and rate-limiting factor in glycolysis. In contrast, fructose rapidly enters the glycolytic pathway at the triose phosphate level. This bypass leads to a more rapid depletion of ATP. The resultant decrease in ATP levels causes a corresponding increase in AMP, signaling a reduced energy status within the cell. The activation of AMPK detects this change in the ATP/AMP ratio. Consequently, the level of Malonyl-CoA, a molecule involved in fatty acid synthesis and appetite regulation, also decreases. All these processes increase food intake, as the body attempts to compensate for the perceived energy deficit. In contrast, ingested glucose has the opposite effect as it exerts an anorexigenic impact after ingestion [18, 32, 89, 98, 99].

## 7. High Fructose Consumption and Its Relationship With Neuroinflammation and Cognitive Dysfunction

The brain and its neuronal function are highly dependent on the mitochondria and its aerobic oxidative phosphorylation to satisfy its energetic needs, as neurons cannot generate energy through glycolysis [100]. Reactive oxygen species (ROS) are byproducts of normal cellular respiration, particularly during oxidative phosphorylation. While ROS are normal and even necessary for some cellular functions, they can be damaging in high concentrations and play a key role in neurodegenerative diseases by their potential to cause oxidative stress in various cells. Therefore, any dysfunction in mitochondrial activity puts the brain at risk of impairment of its functions [100–102]. Additionally, fructose is significantly more reactive than glucose in forming advanced glycation end-products (AGEs), which further contribute to oxidative stress and neuroinflammation. Recent studies have shown that fructose-derived AGEs (Fruc-AGEs) accumulate in hippocampal neurons, triggering RAGE/NF-κB signaling and inducing mitochondrial dysfunction, reactive gliosis, and neuronal impairment—hallmarks of early neurodegenerative processes [103]. Moreover, Fruc-AGEs have been detected in endothelial cells, where they promote oxidative damage and inflammatory gene expression, further supporting their pathological role in brain dysfunction [104]. Some findings even suggest that AGEs can be formed within the intestine before systemic absorption, exacerbating metabolic and neurological consequences [103, 104].

The hippocampus is a crucial brain area in the control of memory and learning activities, and its dysfunction may cause altered cognitive status, evidenced by the presence of hippocampal inflammation as an early event in neurodegenerative diseases and its significant impact on neurological alterations [105–107]. Peroxisome proliferator-activated receptor gamma coactivator-1-alpha (PGC1-α) along with the cytochrome c oxidase subunit II (COX2) are transcriptional factors involved in mitochondrial energy production and biogenesis [108]. In a study conducted by Jiménez-Maldonado et al. [109], they observed that these mitochondrial markers were negatively affected after just 1 week of high fructose intake in the hippocampus, independent of peripheral metabolic alterations such as body weight, obesity, or glucose intolerance. These findings challenge the common belief that brain alterations result from peripheral alterations or after the development of metabolic syndrome, suggesting that fructose-induced mitochondrial dysfunction may be an initial contributor [110, 111]. Furthermore, they highlight that during childhood and adolescence, which are critical periods of neurocognitive development, ingesting a high-fructose diet is especially harmful [110, 111]. It is reported that even 1 week of a high-fructose diet can start changes in the hippocampus [109]. As a response, human research studies in children with neuroimaging have also been conducted and showed that a high-fructose-intake diet can induce changes in the structure and connectivity of the hippocampus [112].

Similarly, Cigliano et al. [110] reported that after 2 weeks of a high-fructose diet, there was an increase of plasma lipopolysaccharide, glial fibrillar acidic protein (GFAP), and TNF-α in the hippocampus of rats without a change in body weight or body adiposity. They reported that the high-fructose diet could trigger neuroinflammation that may take place even before the onset of weight gain or obesity. They state that the high-fructose-diet effects on the hippocampus may be due to the direct effect of fructose in the choroid plexus and microglial cells, as they also express GLUT5 transporter [113, 114]. In their study [110], they also reported high levels of nitro-tyrosine (N-Tyr) and thiobarbituric acid-reactive substance (TBARS) in the hippocampus, which are markers of oxidative damage and neuronal cell death, along with the reduction in the expression of PGC-1α.

Since GLUT5 is widely present in many tissues such as the small intestine, liver, adipose tissue, testis, muscle skeletal tissue, and brain, the absorption and metabolism of fructose can also take place in these organs [41]. A study with mice that were given labeled fructose showed that even in small amounts of fructose administered, it could reach the brain [45]. In these high-fructose-fed rats, the increased levels of uric acid were correlated with an increased fructose metabolism in the hippocampus region. Interestingly, the increased uric acid levels observed in rats fed with fructose were suggestive of an enhancement in fructose metabolism in the hippocampus. Uric acid in the intracellular compartment can induce inflammation, oxidative stress, and activation of NF-kB [115–117]. Specifically, it was shown that uric acid produces inflammation in the hippocampus via the TLR4/NF-kB pathway, which can cause cognitive dysfunction [117]. The relevancy of this study lies in the fact that the increased levels of fructose and uric acid by the fructose diet occurred along with the hippocampal inflammation, and the switch to a control diet reversed all the inflammatory changes and the fructose and uric acid levels [117].

To assess the effects of a high-fructose diet in a younger population and its impact on the developing brain, Mazzoli et al. [118] demonstrated that a high-fructose diet for 3 weeks was associated with increased GLUT5 and fructose in the hippocampus. They also reported high levels of N-Tyr and TBARS and altered redox homeostasis, evidenced by reduced activity in antioxidant enzymes: superoxide dismutase activity (SOD) and glutathione reductase (GSR) [119]. Additionally, all these changes were fully reversed after returning to the control diet, proving that fructose induced alterations at a young age and how a change in diet could reverse those changes.

Interestingly, in a different study conducted by Fierros-Campuzano et al. [120], 60 male Wistar rats were exposed to drinking a solution of 10% fructose for 12 weeks, beginning in their adolescence, and then removing the fructose from the diet. The fructose diet resulted in increased hippocampal inflammatory markers, such as IL-1β and GFAP, and despite removing the diet for 4 weeks, there was still noticeable neuroinflammation, mitochondrial dysfunction, and spatial memory impairment. This indicates that some cortical and hippocampal changes were irreversible even after switching to a control diet. These reported differences might be justified by the time of exposure to fructose.

GLP-1 has been evidenced to show neuroprotective properties in the brain and improve insulin resistance. Glucagon-like peptide-1 receptor (GLP-1R) is expressed in brain areas like the hippocampus, neocortex, and hypothalamus [121–123]. GLP-1R mice have shown impairment in memory, learning, and brain plasticity [124–126]. In this same line, insulin receptor (ISNR) signaling plays a vital role in brain metabolism, plasticity, and neuroprotection, as evidenced in neurodegenerative diseases and neurodevelopmental problems with ISNR deficits [125]. In this context, a study that was conducted in Wistar rats that were given 20% fructose water for 16 weeks [126] showed a statistically significant decrease in ISNR mRNA with a tendency also to decrease GLP-1R mRNA in the hippocampus, independently of the rest metabolic parameter of body weight, fasting plasma insulin and GLP-1, HDL, LDL, and cholesterol which were not affected.

In terms of a more prolonged time phase of a high-fructose diet, a study by Wu et al. [127] showed that high fructose ingestion during 8 months induced impaired memory and learning in rats, a marked decrease in the insulin-signaling pathway, and induced insulin resistance in the hypothalamus. Also, in the experiment, peripheral insulin resistance was correlated with cognitive function. One theory is that presenting hyperinsulinemia for a prolonged period could send signals to downregulate blood–brain barrier ISNRs and decrease the amount of insulin transported to the brain. This correlates with the studies that showed that administering insulin intranasally improves memory, and insulin infusion directly in the hippocampus improves various memory tasks [128, 129]. Additionally, a high-fructose diet can induce the formation of triglycerides, which can cross the blood–brain barrier and induce hippocampal-associated memory alterations [130].

## 8. Conclusion

The increasing prevalence of high-fructose diets poses significant health risks due to the rapid and unregulated metabolism of fructose without feedback inhibition, perpetuating a cycle of continuous food-seeking behavior and upregulation of its metabolic pathways. A prolonged high intake of fructose may cause alterations in the brain related to neurogenesis, insulin signaling, mitochondrial dysfunction, and neuroinflammation. These alterations may be the initial contributors to the development of cognitive impairments and even neurodegenerative diseases, highlighting the urgent need for additional human clinical trials. Such studies are necessary to deepen our understanding of the effects of high fructose consumption with differing effects observed in childhood versus adulthood and according to the duration of exposure to develop targeted awareness and intervention strategies.

## Data Availability

This publication does not present new data. All information included has been sourced from previously published articles and is appropriately referenced. If further details or data are required, they can be provided through reasonable requests to the original authors.
